# Composite Beams Made of Waste Wood-Particle Boards, Fastened to Solid Timber Frame by Dowel-Type Fasteners

**DOI:** 10.3390/ma16062426

**Published:** 2023-03-18

**Authors:** Meta Kržan, Tomaž Pazlar, Boštjan Ber

**Affiliations:** 1 Slovenian National Building and Civil Engineering Institute (ZAG), 1000 Ljubljana, Slovenia; 2 Jelovica hiše d.o.o., 4205 Preddvor, Slovenia

**Keywords:** composite timber beam, box beam, OSB, cement-particle boards, innovative engineered wood product, experimental tests, flexural performance, mechanical fasteners

## Abstract

To increase the sustainability of prefabricated timber buildings and constructions, composite timber beams with “box” cross-sections were developed in collaboration with an industry partner. They were constructed from a solid timber frame and from webs made of residual waste wood-particle boards from prefabricated timber buildings production. The developed beams’ design concepts presented in this paper were governed by architectural features of prefabricated timber buildings, geometrical limitations, available production technology, and structural demand related to various possible applications. The paper presents the results of experimental bending tests of six variations of the developed composite timber beams constructed by mechanical fasteners only. The developed design concept of composite timber beams without adhesives is beneficial compared to glued beams in terms of design for deconstruction and lower VOC emissions. The tests were conducted to study the influence of the following parameters on the beams’ mechanical behavior: (i) web material (oriented strand boards (OSBs) vs. cement-particle boards); (ii) the influence of beam timber frame design (flanges and web stiffeners vs. flanges, web stiffeners, and compressive diagonals), and (iii) the influence of stiffener–flange joint design. Besides the beams’ load-bearing capacities, their linear and non-linear stiffness characteristics were the main research interest. While adding compressive timber diagonals did not prove to significantly increase the stiffness of the beams in the case of cement-particle board webs, it increased their load-bearing capacity by enabling the failure of flanges instead of prior webs and stiffener–flange joints failure. For beams with OSB webs, failure of the bottom flange was achieved already with the “basic” timber frame design, but timber diagonals proved beneficial to increase the stiffness characteristics. Finally, mechanical characteristics of the developed beams needed in structural design for their application are provided together with further development guidelines.

## 1. Introduction

### 1.1. Research Background with Short Literature Overview on Composite Timber Beams

The advantages of engineered wood products were recognized already a century ago; first, timber composite assemblies date back to the 1920s and were developed for use in the aerospace industry [1], while the first composite beams for structural applications in buildings were reported in the mid-1930s in Europe [2]. Glued laminated timber and glued solid timber are nowadays, amongst timber engineered products, the most commonly used to bridge large open spaces with long spans for timber structures in Europe [3]; however, due to the advantages of engineered composite beams compared to solid timber beams, such as significant self-weight reduction, lower material consumption, and a greater possibility of “tailoring” mechanical characteristics, composite beams are a relevant alternative. They are widely used for different structural applications such as roof support girders, floor joists, or headers over openings [2], especially in North America, and their use is expected to grow [4]. 

Many different types of composite timber beams are available on the market differing in the materials used and in the design. Panel web beams are more common than lattice web beams and differ in cross-sections; I-beams (joists), recessed and box beams with two or more webs, etc., can be found [5]. The webs are most often made of hardboard, plywood, or oriented strand boards (OSB). They are, by adhesives or mechanical fasteners, connected to flanges usually made of solid finger jointed or glued laminated timber but can be made of other materials, such as laminated veneer lumber (LVL), too [6]. The connection between the flanges and the web is crucial for the beams’ mechanical behavior as a shear-resistant interface between the components [7]. In most factory-type productions, it consists of a continuous glue-line of rigid adhesive between the members, which enables complete interaction between them [6]. On the other hand, connections with mechanical fasteners, such as nails or screws, which are, compared to adhesively bonded connections, more desirable from the design for disassembly and environmental points of view, are characterized as semi-rigid when used. In such connections, a lateral slip between the joint members occurs at lateral loading and the stiffness and deformations of the composite timber beams depend highly on the interaction between the flanges and the web, namely the connection’s slip modulus [7]. As such shear connections generally allow only partial composite action between the individual components of the beams, the mechanical behavior of such members is quite complex and, due to the interlayer slip, is difficult to predict [8]. Furthermore, depending on the web material and the beam geometry, web stiffeners should be included in the design to prevent web buckling [9] and to enable the beams favorable failure modes, which utilize materials in the greatest manner.

Past and current studies for the development of engineered wood products in the field of timber composites spanning structural elements, such as beams or joists, focus on a variety of aspects of their behavior related to different issues, such as methods for analysis or design, behaviors under short-term load, the influence of flange and web characteristics (material, orientation, reinforcement, openings, web butt joints, flange–web joints) on the mechanical behavior, the impact of moisture content, and various aspects of long-term behavior. A comprehensive literature overview up to 1990 is presented in Leichti et al. [10], while numerous later research studies on timber joists are summarized in Chen et al. [11]. Timber composites on the structural level often include cement-based materials; timber–concrete composites (TCCs) commonly used for timber–concrete beams and floor or bridge deck systems, for example, are widely used [12,13]. The higher density of cementitious materials is beneficial for structural vibrations, fire, and acoustic performance. To overcome the major disadvantages of cementitious materials, i.e., low tensile and flexural strength, high-performance cementitious materials are being developed [14], which are low-carbon and ecological and increase the potential of development of more sustainable timber–cementitious material composites both on structural and material scales.

Nowadays, new demands for composite timber beams/girders and the need to evaluate various aspects of their performance arise from ever-more-demanding architectural designs not only in terms of longer spans or higher loads but also for the consequential need for better mechanical characteristics. Their specific use and inclusion in the broader building system are also relevant, with characteristics such as thermal conductivity or installation flow governing the design as well. An important aspect nowadays is also sustainability, which introduces additional features to the design of engineered products such as life cycle analysis (LCA) for reducing greenhouse gas emissions [15,16], for adaptability [17], and for disassembly in light of the circular economy [18].

### 1.2. Motivation for Development of the Presented Designed Composite Timber Beams

**Figure 1:**
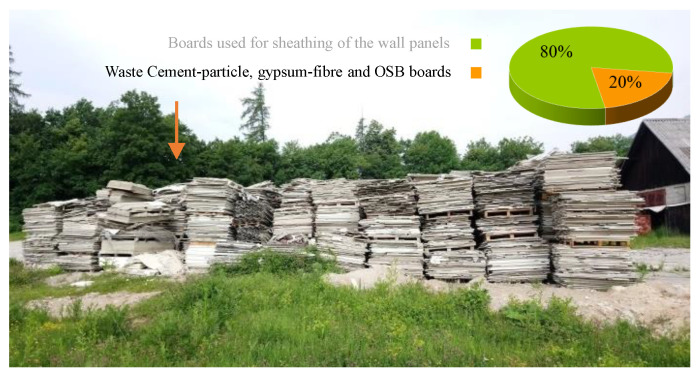
Residual waste boards arising from light-frame timber panels production in the manufacturing of prefabricated timber buildings.

A Slovenian manufacturer of prefabricated timber buildings uses prefabricated light-frame timber wall panels as vertical load-bearing elements. For their production, the manufacturer first tailors solid or glued laminated timber lengthwise, from which a frame is assembled. To ensure the shear load-bearing capacity and rigidity, the frame is sheathed with various types of wood-based or gypsum-fiber boards. A substantial amount of residual waste is created in this production with cutting of sheathing boards that, due to their size, cannot further be used in the regular production of wall and floor structural elements. In the case of this specific manufacturer, approx. 20% of the purchase quantity of boards, i.e., oriented strand boards (OSBs), cement-particle boards, and gypsum-fiber boards (Figure 1), must therefore be properly disposed as residual waste.

On the other hand, in practice, glued laminated timber and often also steel girders are used in the construction of prefabricated timber residential houses due to modern architectural designs with large and open spaces. Due to static requirements, they are also used in positions of larger openings.

For these reasons, in collaboration with an industry partner, i.e., a manufacturer of prefabricated timber buildings, design concepts for composite timber beams were developed, where waste sheathing boards could efficiently be utilized and such beams used in prefabricated timber buildings (or elsewhere). The use of the designed beams instead of glued laminated timber or steel beams would contribute to increased sustainability even more. While the use of waste boards dictates the beams concept design with a limited length of the boards for the webs, the beams’ geometry is highly dependent and limited on the beams’ designated use applications in the prefabricated timber buildings already designed.

A peculiarity of the presented developed beams is the use of waste cement-particle boards for the webs. Composites of timber and cementitious materials have many advantages such as improved fire, weather, and insects/fungi characteristics, but the use of cement-particle boards for structural application is still not common: it is found in the literature [19] or in product catalogues of manufacturers only for light-frame timber or steel wall panel constructions ([20]). 

### 1.3. Research Study

This paper presents the developed design concepts for composite timber beams, made of solid timber frames and waste sheathing boards attached to a timber frame by mechanical fasteners only, without adhesives. As the mechanical behavior of the developed composite beams depends on various parameters with high standard deviation and non-linear characteristics (such as slip modulus of board-timber connections), an experimental study was conducted to evaluate and study their bending performance. 

Altogether, 18 bending tests on the developed real-size composite timber beams were conducted to experimentally evaluate the bending performance of different beam designs and to compare their behavior to each other. With systematic variations in design parameters in the tested girders, the influence of the type of boards used for the web, the influence of the timber frame design, and the influence of the flange–stiffener joints on the mechanical behavior of the developed beams are analyzed and discussed in this paper. The designed beams’ characteristic bending resistance and stiffness values, which can be used in structural design for their application, are finally presented together with guidelines for further research for development.

## 2. Materials and Methods

### 2.1. The Developed Design Concept of the Composite Timber Beams with Material Characterization and Structural Details

#### 2.1.1. The Developed Design Concepts of the Composite Timber Beams

**Figure 2:**
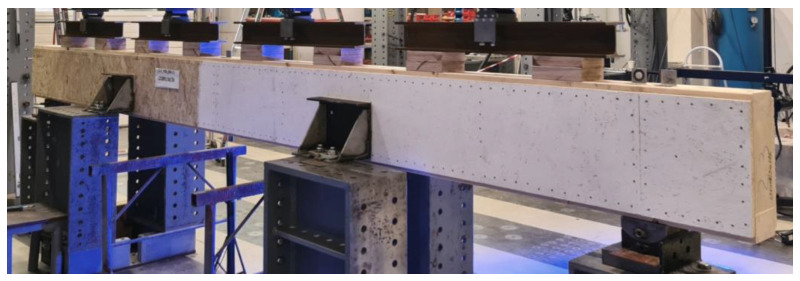
Composite timber beam made of waste OSB boards, fixed to a glued laminated timber frame (flanges and stiffeners) by nails.

The presented developed designs were based on the materials used in the production of prefabricated timber buildings of the industry partner and the technical feasibility of the beams’ production in their premises with the technology available. To develop an efficient design concept suitable for the industry partner by considering not only the mechanical performance of the beams but also the time and cost for their production, various design concepts were evaluated and experimentally tested. Different variations of the developed and experimentally tested composite timber beams are summarized in Table 1, while a variation of the composite timber beam with OSBs constructed for experimental testing is presented in Figure 2.

The basic and simplest variations of the composite timber beams were constructed of a timber frame made of glued laminated timber flanges and stiffeners (vertical studs), to which waste wood-particle boards, either cement-particle boards or OSBs, were attached by mechanical fasteners, metal staples or nails, respectively (variations labeled 1.1-V1-CPB and 2.1-V1-OSB in Table 1). 

**Figure 3:**
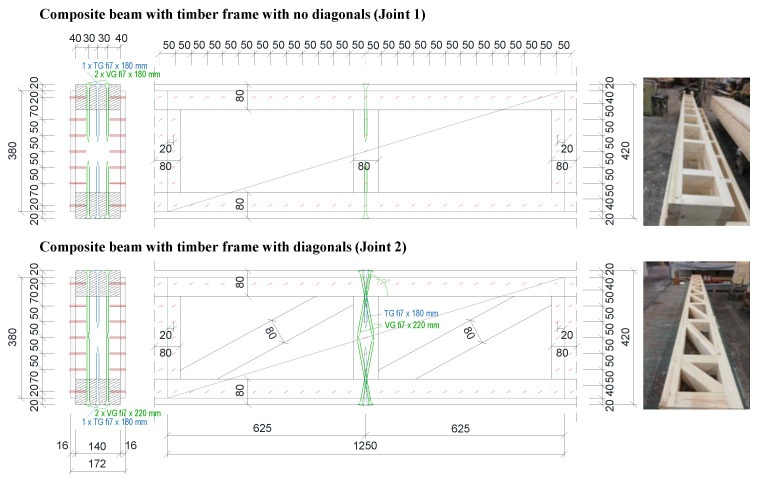
Cross-sections and side view cutouts (along longitudinal axes) of beams with cement-particle boards with details of stiffener–flange joints (in mm) and photos of constructed timber frames for beams with no diagonals (above) and beams with diagonals (bottom).

To achieve a higher stiffness of the beams, glued laminated timber diagonals were for beams with both types of boards added to the timber frame (variations 1.3-V2D-CPB, 1.5-V1D-CPB, and 2.3-V2D-OSB; label “D” in beam variations names). Because of the different internal force distribution in beams with added compression diagonals, the basic stiffener–flange joint (Joint 1, label “V1” in beam variations names) was changed for these variations (Joint 2; label “V2” in beam variations names, Figure 3). For comparison, the mechanical behavior of a composite beam with added diagonals but with the basic stiffener–flange joints was tested on one beam variation with added diagonals as well (1.5-V1D-CPB). To analyze the behavior of the timber frame with diagonals alone (without the webs), beams with diagonals and no web panels were tested as well (0.1-V1D).

The costs of production are not within the scope of the paper, but it can at this point be mentioned that besides the extra material for diagonals, the production of beams with diagonals is considerably more time-consuming than of beams with the “basic” frame.

The height of the developed beam variations chosen to be tested was equal for all variations and determined considering the maximum available height over openings in the architectural design of the manufacturers-prefabricated timber buildings at the time of the composite timber beam concept development. The height of the beams could, however, with new adapted building designs, be increased. The width of the beams was governed by the common wall panel design. All the experimentally tested composite timber beam variations had the same dimensions of the timber frame elements.

#### 2.1.2. Materials Characterization

All components of the timber frame for the developed composite timber beams were constructed from glued laminated “duo” structural timber of strength class GL 24 h.

Both types of waste boards used, i.e., OSB and cement-particle boards, were engineered wood products, commercially available on the market. The OSB boards are produced as three-layered synthetic resin-bonded wood-based panels made of side-by-side oriented strands (microveneers) according to EN 300:2006 [21]. The OSB waste boards used for the composite beams are appropriate for structural use in humid conditions (OSB 3, EN 13986:2005 [22]). The waste cement-particle boards used for the beams are made in a flat press from reduced wood material, wood chips, and with the addition of hydraulic binder (Portland cement) and chemical additives. The density of the boards is 1400 kg/m^3^ ± 100 kg/m^3^, making them applicable for lightweight construction and having some beneficial mechanical and physical characteristics of cementitious materials in comparison to wood, such as improved fire, weather, and insects/fungi characteristics. They are also formaldehyde-free [20].

The mechanical tests of the boards were not conducted, as sufficient information on the mechanical characteristics to develop the design concept of the composite beams is provided in declarations of performance [23,24]. The mechanical characteristics of the boards in their plane, specified in the technical data sheets of the manufacturer, for cement-particle boards, are as follows: flexural strength *f*_m, k_ = 9.0 MPa, tensile strength *f*_t, k_ = 2.5 MPa, shear strength *f*_v, k_ = 6.5 MPa, compressive strength *f*_c, k_ = 11.5 MPa, and modulus of elasticity *E*_m, mean_ = 4500 MPa; for OSB boards: flexural strength *f*_m, k_ = 14.8 MPa, tensile strength *f*_t, k_ = 7.4 MPa, shear strength *f*_v, k_ = 6.8 MPa, compressive strength *f*_c, k_ = 14.8 MPa, and modulus of elasticity *E*_m, mean_ = 3800 MPa.

#### 2.1.3. Structural Details

**Figure 4:**
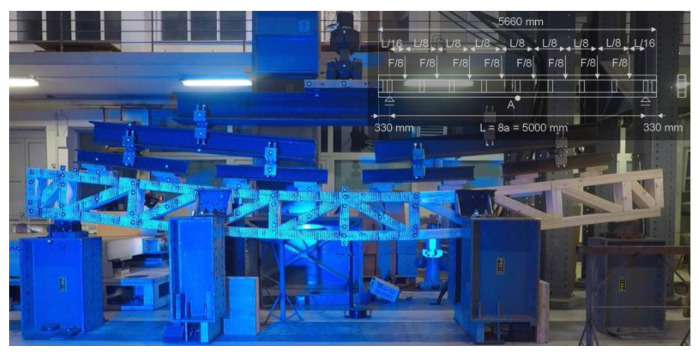
Test setup for bending tests of composite timber beams (variation 0.1-V1D; timber frame with compressive diagonals and no web panels).

The timber frame flanges, web stiffeners (vertical studs), and diagonals (when used) all had 140/80 mm (width/height) dimensions. In all variations, the web stiffeners were arranged at a 625 mm distance (Figure 3), which was also the module for vertical studs in light-frame timber panels. The distance between the end stiffeners was adjusted to the length of the beams (Figure 3 (right)). For beam variations with diagonals, the diagonals were oriented in the direction to be loaded in compression (Figure 3 (below), Figure 4) and were therefore only inserted in the timber frame (no mechanical connection).

The developed composite timber beams had cement-particle boards of 16 mm thickness or OSBs of 22 mm thickness attached to both sides of the timber frame as webs. The length of the boards was, except at the beams ends, 1250 mm, which is a multiplier of the 625 mm distance module between stiffeners and also the width of the whole-size wood-particle boards that the manufacturer uses and is thus the most common length of waste pieces. The height of the boards corresponded to the height of the beam reduced by 40 mm to allow a 20 mm displacement of the boards, relative to the flanges’ outer edges on both sides. The boards on one side of the beam were longitudinally offset by 625 mm relative to the other side along the beam with the length of end boards adjusted to the beams’ length. The cross-section and longitudinal side view cutouts typical for beams without (above) and with diagonals (below) are presented in Figure 3.

Both board types were fixed to the timber frame (flanges and stiffeners) by dowel-type mechanical fasteners along the boards’ perimeter at a 50 mm longitudinal spacing distance and 20 mm distance from the boards’ edge, and along the center line of the intermediate stiffener (Figure 2 and Figure 3). The cement-particle boards were fastened to the timber frame by 2.0 × 11.76 × 50 mm metal staples with a characteristic tensile strength of galvanized steel for staples equal to *f*_u, k_ = 900 MPa and with a layer of thermo-adhesive coating applied on the staple legs [25] to increase the fastening. The OSBs were fixed to the timber frame by 2.5 × 65 mm nails with a characteristic tensile strength of galvanized steel for nails equal to *f*_u_,_k_ = 600 MPa and a layer of thermo-adhesive coating applied on the shank and point [26]. To study the shear behavior of the cement-particle board–timber connections by staples, preliminary experimental tests of connections were conducted [27] where it was confirmed that a 50 mm spacing distance between the staples was suitable for the cement-particle boards despite the higher distance recommendations by the board manufacturers (e.g., [20]). The experimental test results also proved a higher characteristic lateral load-bearing resistance than the one calculated according to EC5 (EN 1995-1-1:2004: Eurocode 5: Design of timber structures—Part 1-1: General—Common rules and rules for buildings [28]). As already mentioned, for the deformations of the developed beams, the slip modulus of the web–timber frame connection (stiffness *K*_ser_) is more important than the shear load-bearing resistance. The results showed a similar slip modulus obtained according to experimental tests and calculated according to the EC5 standard.

Preliminary numerical studies have shown a higher load-bearing capacity for beams, in which shear forces are not transferred only by web–timber frame connections but through stiffener–flange joints as well. Thus, all variations of beams had stiffeners fixed to flanges with three timber screws at each joint. The beams with “basic” timber frames had stiffeners fixed to flanges by a partially threaded screw (TG) 8 × 180 mm [29] in the middle and two fully threaded screws (VG) 7 × 180 mm [29] at the sides with 30 mm distances between the screws (Joint 1 in Figure 3). Beam variations with diagonals had, apart from one variation (1.5-V1D-CPB), stiffeners attached to flanges by a joint with an increased withdrawal capacity (Joint 2 in Figure 3) compared to the basic joint (Joint 1); screws at the side were replaced by two VG 7 × 220 mm screws [29] inserted at an angle of approximately 10° to the vertical axis.

### 2.2. Experimental Tests

The experimental campaign consisted of bending tests of five variations of developed beams of 420 mm height, where the type of boards and corresponding board–timber connections, the presence of timber diagonals, and the type of stiffener–flange joints were systematically varied (Table 1). For each variation, three beams were tested. Among the three specimens for each tested beam variation, one was tested with an extended loading protocol to analyze the creep behavior. To evaluate the contribution of webs to the beams’ resistance and stiffness, three timber frame beams with diagonals and no webs were tested.

The beams for experimental testing were, besides the geometrical requirements of the industry partner, designed considering the test setup and loading protocols’ guidelines in standards for similar structural elements, namely EN 13377:2002 (Prefabricated timber formwork beams—Requirements, classification and assessment [30]), EN 408:2010 (Timber structures—Structural timber and glued laminated timber—Determination of some physical and mechanical properties [31]), and EN 595:1995 (Timber structures—Test methods—Test of trusses for the determination of strength and deformation behavior [32]). The test setup is presented in Figure 4. The beams were simply vertically supported with a 5000 mm distance between the supports and 330 mm overhang length (more than half of the lattice beam module overhang length prescribed in EN 13377:2002). The length between the supports was chosen such that the stiffener and support positions would coincide. To prevent out-of-plane buckling, the beams were laterally supported in two positions. Distributed load was applied to the beams at 8 equally spaced positions in accordance with EN 13377:2002. The loading protocol based on EN 595:1995 had several phases:—1st loading phase with loading in the elastic range (loading, 30 min constant load, unloading);—*2nd loading phase with constant load for creep analysis (*this phase was conducted for one of the three test specimens for each beam variation: loading, 18 h constant load to force where *l*_0_/300 mid-span vertical displacement is achieved, unloading);—3rd loading phase with loading to failure.

During the tests, displacements and deformations over approximately half the length of the beam were measured by the optical measuring system through digital image correlation (DIC). Discrete targets were put on beams at points of interest to measure displacements, while the front surface was colored with a pattern to monitor the deformations and damage during the test. The vertical displacements of the beam at mid-span and at the support were measured with linear variable differential transducers (LVDTs), except at very large deformations where the LVDT at mid-span was removed to prevent its damage.

## 3. Results and Discussion

### 3.1. Damage Mechanisms

**Figure 5:**
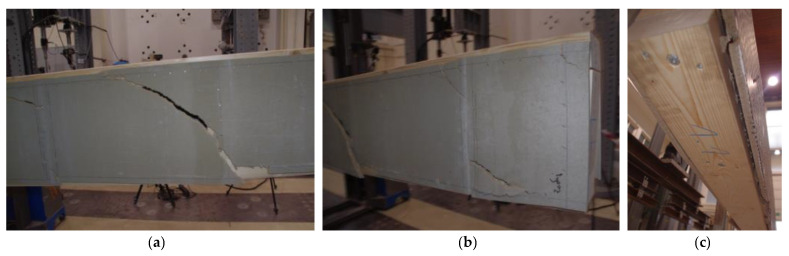
Damage mechanisms obtained for beams with cement-particle boards: (**a**) Tensile cracks of the cement-particle board and block shear failure of the board (right half of board); (**b**) Block shear and row shear failure of the boards in various locations; (**c**) Large deformations of the web–timber frame connections with more visible pull-out of the staples at beam’s end.

**Figure 6:**
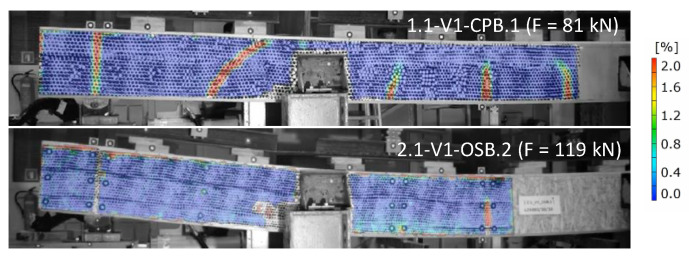
Deformations measured in the boards with optical measuring system before the beams’ failure for beam without diagonals with cement-particle boards (**top**) or with OSBs (**bottom**).

In the conducted experimental tests, different damage mechanisms were obtained for different variations of beams mainly in dependence of the type of boards used. In the case of beams with cement-particle boards, several cracks in the boards occurred due to exceeded tensile stresses in the boards (Figure 5a) and due to block shear failure of the boards along the connections (Figure 5b). At the beams ends, large displacements in the boards relative to timber flanges were observed, accompanied with an evident pull-out of the staples from the timber frame (Figure 5c). On the other hand, failure of beams with OSBs occurred without cracks in the boards due to their better mechanical characteristics, i.e., higher tensile and flexural strength. The difference was evident from the comparison of deformations measured before the failure with the optical measuring system (DIC) for the beam with the “basic” timber frame design and cement-particle boards and for the beam with the “basic” timber frame design and OSBs in Figure 6.

**Figure 7:**
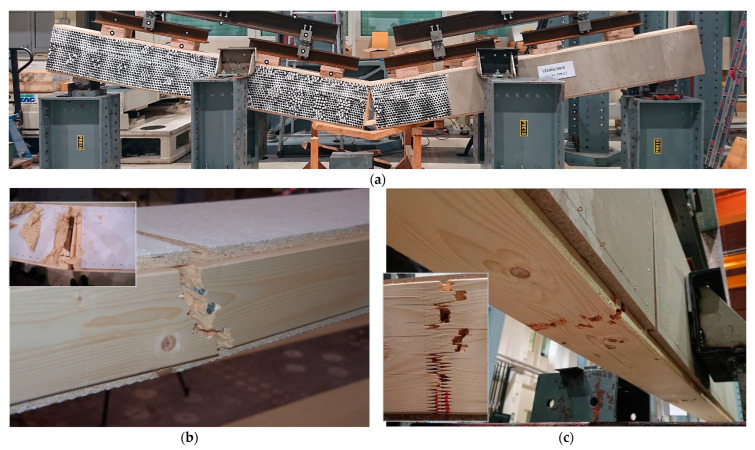
Typical failure mechanism of beams with cement-particle boards with failure of the bottom flange: (**a**) Failure of the beam with obtained damage of the boards; (**b**) Failure of the bottom flange at stiffener–flange joint with stiffener splitting (smaller photo in the corner); (**c**) Failure of the bottom flange at finger joint of the lower lamella (specimen 1.3-V2D-CPB.2*) with approximately 30% of the finger joint failing in glue-line (smaller photo in the corner).

For both beams with cement-particle boards and with OSBs, the beams collapsed due to failure of the bottom flange. The final failure of the beam with cement-particle boards is presented in Figure 7a. The failure of the flange often occurred in the stiffener–flange joint (Figure 7b), but this was not the rule. In such cases, the stiffener often split (Figure 7b). In the joints, the resistance of the flange was, in general, lower because of the reduced cross-section due to connectors. However, as the timber was an inhomogeneous material, of which local mechanical characteristics depend also on the presence of knots and their size and position, rate of growth, slope of grain, etc., some other cross-sections may be more critical. The weakest cross-section was often found where the lamellas were jointed by finger joints; in one third of the specimens (not counting beams without webs), the failure occurred at finger joints, usually of the lower lamella (Figure 7c).

**Figure 8:**
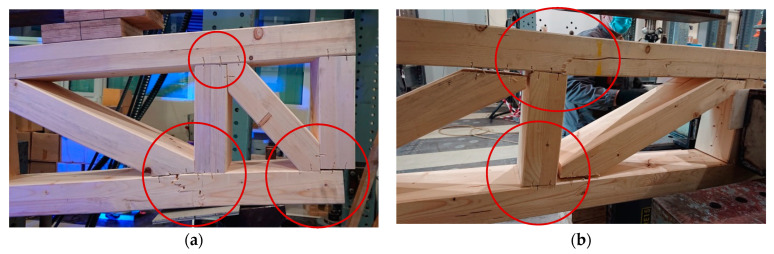
Typical failure and damage mechanisms of beams with no web panels: (**a**) Failure of the bottom flange at vertical stud–flange joint and large deformations of the vertical stud–flange joints; (**b**) Shear failure of the upper flange at vertical stud–flange joint and large deformations of the vertical stud–flange joints.

In the case of timber frame beams with no webs, large deformations occurred in the vertical stud–flange joints and were more evident around the supports (Figure 8), which is where the failure of the beams also occurred; the failure of the bottom flange at the vertical stud–flange joint at the support can be seen in Figure 8a, while Figure 8b shows the failure of the upper flange at the vertical stud–flange joint.

### 3.2. Bending Load-Bearing Capacity, Stiffness, Creep, and Relaxation

**Figure 9:**
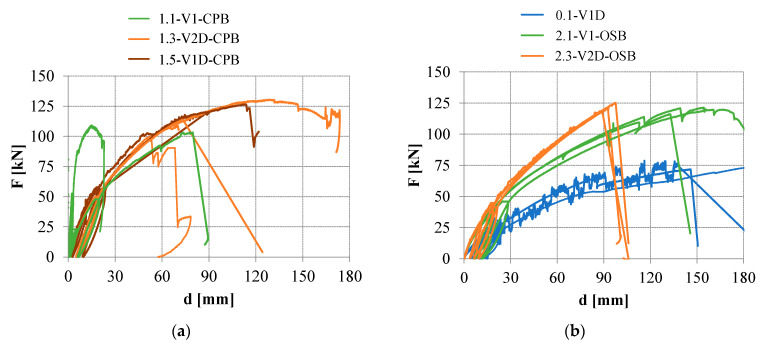
Diagrams of vertical forces obtained in dependence of the mid-span vertical displacements: (**a**) For beams with cement-particle boards; (**b**) For beams with OSBs and beams with no web panels.

The diagrams of vertical force in dependence of the vertical displacement of the beam at mid-span, *d*, presented in Figure 9, clearly show the influence of the varied parameters on the bending load-bearing capacity as well as the stiffness of the beams obtained. Figure 9a displays the results of beam variations with cement-particle boards where, besides the beam design, the influence of loading rate on the stiffness is also evident (1.1-V1-CPB specimen), while Figure 9b displays the results of beam variations with OSBs and beams with no web panels. In Table 2, most relevant results for individual tests are presented, namely the maximum force achieved, *F*_max_; the vertical displacements of the beam at mid-span at maximum load-bearing capacity, *d*_Fmax_, and at 10% of the load-bearing capacity, *d*_0.1_; the elastic stiffness, *k*_0.1–0.4_, i.e., secant flexural stiffness between 10% and 40% of the load-bearing capacity; plastic stiffness, *k*_0.4–0.9_, i.e., secant flexural stiffness between 40% and 90% of the load-bearing capacity (both stiffness values presented are for the beams’ loading to failure test phase).

**Figure 10:**
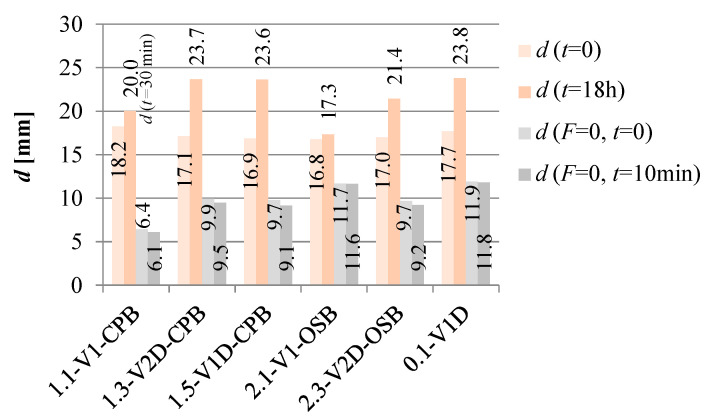
Beam vertical mid-span displacements at the beginning and the end of 18 h constant loading (*d* (*t* = 0) and *d* (*t* = 18 h), respectively), after unloading *d* (*F* = 0, *t* = 0), and after 10 min relaxation of the beams *d* (*F* = 0, *t* = 10 min).

The vertical mid-span displacements for beams tested with the loading protocol with an additional loading cycle under a constant 18 h load are presented in Figure 10: namely, displacements at the reached assigned constant force for 18 h loading, *d* (*t* = 0); at the end of 18 h loading, *d* (*t* = 18 h); after unloading, *d* (*F* = 0, *t* = 0); 10 min after unloading, *d* (*F* = 0, *t* = 10 min). It must be noted that specimen 1.1-V1-CPB.2* was loaded for 18 h up to a very small displacement, which cannot be compared to other tests; therefore, the column presented in Figure 10 for the mentioned test shows the displacement of the beam under comparable loading (load for *l*_0_/300 displacement) after 30 min, *d* (*t* = 30 min), and not 18 h loading.

The measured increment in mid-span displacements due to the creep between the end and beginning of the constant 18 h loading was between 4.5 and 6.8 mm. The first result corresponds to the beam with OSB webs and diagonals, while the second corresponds to the beam with cement-particle board webs, diagonals, and weaker stiffener–flange joints. The limit displacements presented 26% and 43% of achieved elastic displacements (defined as displacement at 40% of the load-bearing capacity), respectively. After unloading the force, displacements ranging from 9.7 to 11.9 mm remained in the beams, which then, after 10 min, decreased on average for 0.4 mm.

### 3.3. Comparison of the Results and Discussion

Comparisons show that the design concept with compressive diagonals helped to increase the bending capacity of beams with webs made of cement-particle boards, as it reduced the tensile forces in the boards under equivalent vertical beam loading, enabling failure of the boards at higher beam loading and, by this, achieving the bending load-bearing capacity where the capacity of flanges is fully utilized. For beams with OSB panels, diagonals did not increase the load-bearing capacity, as tensile-bending failure in the bottom flanges occurred without any cracks of the OSBs. The average load-bearing capacities of both design concepts of beams with OSBs—with and without diagonals—were similar with less than 2% of difference. The mean results of mechanical characteristics obtained for specific beam variations are, together with coefficients of variation (CV), summarized in Table 3.

Interestingly, in the case of beams with cement-particle boards and diagonals, a better average load-bearing capacity was achieved for beams with weaker stiffener–flange joints (beam variation 1.5-V1D-CPB). The result can be explained by the small number of specimens and high standard deviation of the results for beams with stronger stiffener–flange joints (beam variation 1.3-V2D-CPB), the cause of which is that in two specimens, the flange failed at the finger joint of the outer lamellas, with one (specimen 1.3-V2D-CPB.2*) among them with approximately 30% of the finger joint failing in the glue-line (Figure 7c).

In the case of beams with OSBs, the diagonals, however, proved to be favorable in terms of stiffness, as their average elastic stiffness *k*_0.1-0.4_ was 47% higher than for beams without diagonals. However, it was still, on average, almost 20% smaller than for beams with cement-particle boards. The lower stiffness of beams with OSBs in comparison to beams with cement-particle boards can mainly be attributed to the higher slip modulus *K*_ser_ of the cement-particle board–timber stapled connections in comparison to OSB–timber nailed connections. If the slip moduli *K*_ser_ of the two selected board–timber (with nails or staples) were similar, the difference in elastic stiffness of the two beam variations would be smaller, as the cement-particle boards used for the construction of beams were thinner but had an 18% higher modulus of elasticity in comparison to the OSBs used. The evaluation of *K*_ser_ for the two used connections according to EC5 [28] suggests that the used cement-particle board–timber connection with staples had a 15.5% higher *K*_ser_ than the used OSB–timber connection with nails. The experimental results of shear tests of OSB–timber connections with nails and staples by Seim et al. [33], in which board thickness and mechanical fasteners were systematically varied, proved, on average, a 55% higher *K*_ser_ for connections with 2.0 mm diameter staples than for connections with 2.5 mm diameter nails in the case of 18 mm thick OSBs, suggesting that the difference between the actual stiffnesses of the connections by staples and nails could be greater than that evaluated according to EC5.

The results of beams with cement-particle boards also strangely showed a higher elastic stiffness for beams without diagonals than for beams with diagonals. According to numerical analyses, the diagonals increased the stiffness of the beams (if the mean values of mechanical characteristics for the built-in materials and *K*_ser_ for the cement-particle board–timber connections according to the results obtained in experimental tests [2] were considered). The smaller stiffness in the case of beams without diagonals may be ascribed to a small number of samples, as the difference in the obtained average stiffness of both tested beam variations was relatively small, but the coefficient of variation for beams without diagonals was significantly larger. For beams without diagonals, the scatter of the results was much higher than for beams with diagonals, again, because the beam stiffness depends to a greater extent on the board–timber connection slip modulus. For cement-particle board–timber connections, *K*_ser_ most likely had a higher statistical dispersion than for the OSB–timber connections due to the more dispersed mechanical characteristics of cement-particle boards caused by the different age and cement hydration of the beams.

It can also be noted that for some beams with cement-particle boards and diagonals, a residual load-bearing capacity was observed (see Figure 9a). This can be explained by the redistribution of internal forces to diagonals after achieving the load-bearing capacity, defined by failure of the boards.

In order to use the developed beams in buildings and to confirm their safe use within the analysis for structural design, characteristic bending resistances, *M*_y,k_, were for the different beam variations determined (Table 4). They were calculated following the guidelines in EN 1990 [34] from the mean bending resistances, *M*_y,m_, and corresponding coefficients of variation, *CV*, obtained from experimental tests according to Equation (1). Coefficient *k*_n_ was presumed in dependence of the number of samples as given in the code for the 5% characteristic fractile and a normal distribution with an unknown coefficient of variation. Furthermore, from the experimentally obtained mean beam elastic stiffness (*k*_0.1–0.4_), effective elastic stiffness, *EI*, was determined (Table 4). The analytical formula for calculation of the mid-span displacement of the simply supported beam with span length *l*_0_ subjected to uniformly distributed loading was considered (Equation (2)). The calculated effective elastic stiffness can be used for the evaluation of displacements under various loading and boundary conditions.

(1) $$M_{y,k}=(1-k_{n}\cdot CV)M_{y,m}$$

(2) $$EI=\frac{5\cdot k_{0.1-0.4}\cdot {l_{0}}^{3}}{384}$$

The calculated characteristic bending resistances confirmed the highest characteristic resistance of composite beams with OSB boards; values for beams with and without diagonals were within less than 1% of difference. Characteristic bending resistances were for beam variations with cement-particle boards with, significantly, almost 30% lower values for beams with cement-particle boards without diagonals compared to average values of the two beam variations with the cement-particle boards and diagonals tested. The reason for this difference was the high coefficient of variation obtained from tests of beams without diagonals.

For the beams with the longer experimental loading protocol, the displacement after unloading of the 18 h load and 10 min relaxation amounted for various tested composite timber beam design concepts to between 39% and 42% of the final (instantaneous and creep) displacement after 18 h loading. In static analyses and structural design for applications in buildings, the deformation due to creep should be considered in serviceability checks.

## 4. Conclusions

### 4.1. Summary of Research and Development Study with Main Results

The paper presents the developed detailed design of composite timber beams made of solid timber frames and residual waste wood-particle boards (cement-particle or OSB), constructed only by mechanical fasteners and no adhesives, for applications in prefabricated timber buildings to increase their sustainability and added value for their manufacturer.

The presented results of experimental bending tests, conducted on different variations of the designed real-size composite timber beams, prove their potential to be used for structural applications. From the presented study, the following main conclusions can be drawn:

- For beams with the “basic” timber frame (with flanges, stiffeners, and no diagonals), beams with OSB webs prove a higher load-bearing capacity than beams with cement-particle board webs. The reasons for this are better mechanical characteristics of OSBs compared to cement-particle boards, which enable the beneficial failure mechanism of beams with OSBs with no cracking of the webs, allowing the beams to realize their full bending resistance potential with failure of the bottom flange.
- The use of 16 mm cement-particle boards is in comparison to the use of 22 mm OSBs (and both corresponding connections) beneficial in terms of achieving higher bending stiffness. A higher stiffness can be attributed to the greater slip modulus of the used cement-particle board–timber connections by staples than OSB–timber connections by nails.
- Adding compressive diagonals to the timber frame enables the beams with cement-particle boards to achieve a similar bending load-bearing capacity as obtained for beams with OSBs. For the latter, the diagonals increase the bending stiffness.
- For the tested height of the beams and the production technology of the manufacturer available, the load-bearing capacity increase for beams with cement-particle boards or the bending stiffness increase for beams with OSBs in the case of added timber diagonals does not justify the additional costs for their production.

### 4.2. Possible Applications and Future Development

The conducted experimental tests provide data for the structural design of the developed composite beams without adhesive. The developed structural element can, as designed, be used for indoor applications, where its mechanical characteristics meet the requirements of the specific building design and use (confirmed with static analyses according to current technical standards for the structural design of buildings, such as Eurocodes). However, the experimental results indicate relatively small design loads to satisfy the serviceability limits due to their low stiffness. For more demanding structural applications, the stiffness of the beams should be increased. As the stiffness of the presented beams is limited through the slip modulus of the board (web)–timber frame connections and the distance between the fasteners is at its upper limit, the only possibility to increase the stiffness is by fixing the boards to the timber frame by adhesive. While adhesives for structural bonding of timber and OSB boards are available on the market, one of the challenges would be to find and confirm an appropriate adhesive to bond timber and cement-particle boards for structural application. The fire resistance of the beams has not yet been tested; therefore, application of the beams is currently possible only for small residential buildings, such as single-family houses.

## Figures and Tables

**Table 1:** Developed and tested composite timber beam variations.

Beam Variation	Type of Boards	Board–Timber Connection	Stiffener–Flange Joint	Timber Frame Diagonals	No. of Specimens	Loading Protocol *
0.1-V1D	/	/	Joint 2	Yes	3	a, a, b
1.1-V1-CPB	CPB	Staples	Joint 1	No	3	a, a, b
1.3-V2D-CPB	CPB	Staples	Joint 2	Yes	3	a, a, b
1.5-V1D-CPB	CPB	Staples	Joint 1	Yes	3	a, a, b
2.1-V1-OSB	OSB	Nails	Joint 1	No	3	a, a, b
2.3-V2D-OSB	OSB	Nails	Joint 2	Yes	3	a, a, b

* The loading protocol labeled “a” consists of elastic and failure loading phases (1st and 3rd loading phase, respectively), the loading protocol labeled “b” consists of the elastic loading phase, constant load phase, and failure loading phase (1st, 2nd, and 3rd loading phase, respectively).

**Table 2:** Results of experimental bending tests.

Beam Variation	Specimen No.	*F*_max_ (kN)	*d*_Fmax_ (mm)	*d*_0.1_ (mm)	*k*_0.1–0.4_ (kN/mm)	*k*_0.4–0.9_ (kN/mm)
0.1-V1D	1	71.7	145.9	4.1	1.52	0.43
	2 *	74.0	185.8	7.7	1.30	0.33
	3	78.7	116.1	10.3	1.49	0.45
1.1-V1-CPB	1	83.4	49.5	1.6	4.29	1.66
	2 *	109.1	15.0	0.5	29.5	9.11
	3	103.7	79.7	2.7	3.40	1.30
1.3-V2D-CPB	1	130.4	128.3	3.4	5.00	1.09
	2 *	99.7	53.1	2.3	4.96	2.23
	3	108.7	69.9	2.5	4.47	1.58
1.5-V1D-CPB	1	113.8	73.3	2.9	3.32	1.47
	2 *	126.5	113.5	2.9	3.72	1.37
	3	121.2	90.0	2.7	3.63	1.38
2.1-V1-OSB	1	121.8	154.4	3.4	5.57	0.77
	2 *	120.2	167.1	14.2	4.47	0.69
	3	116.1	132.8	3.2	4.84	1.67
2.3-V2D-OSB	1	125.1	97.0	3.2	3.36	1.08
	2 *	118.1	88.9	3.1	3.72	1.20
	3	121.7	92.5	3.4	4.02	1.15

* Tests with extended loading protocol (loading protocol “b” with 2nd loading phase).

**Table 3:** Average results for different variations of beams with CV for each parameter in brackets.

Beam Variation	*F*_max_ (kN)	*d*_Fmax_ (mm)	*d*_0.1_ (mm)	*k*_0.1–0.4_ (kN/mm)	*k*_0.4–0.9_ (kN/mm)
0.1-V1D	74.8	149.3	7.3	1.44	0.40
	(3.9%)	(19.1%)	(34.6%)	(6.9%)	(13.5%)
1.1-V1-CPB *	98.7	64.6	1.6 *	3.85*	1.48 *
	(11.2%)	(23.3%)	(53.6%)	(11.6%)	(12.3%)
1.3-V2D-CPB	112.9	83.7	2.7	3.56	1.33
	(11.4%)	(38.5%)	(18.0%)	(4.8%)	(35.0%)
1.5-V1D-CPB	120.5	92.3	2.8	3.70	1.41
	(14.3%)	(17.9%)	(4.6%)	(7.3%)	(3.4%)
2.1-V1-OSB	119.4	151.4	6.9	2.03	1.05
	(2.0%)	(9.3%)	(73.9%)	(28.7%)	(42.6%)
2.3-V2D-OSB	121.6	92.8	3.2	2.98	1.14
	(2.4%)	(3.6%)	(4.5%)	(4.0%)	(4.0%)

* Specimen 1.1-V1-CPB.2 is due to different loading protocol not considered in the presented average results.

**Table 4:** Mean and characteristic bending resistance and elastic bending stiffness for the tested beam variations.

Beam Variation	*M*_y,m_ (kNm)	*M*_y,k_ (kNm)	*EI* (kNm^2^)
0.1-V1D	46.8	41.0	2341
1.1-V1-CPB	61.7	39.9	6260
1.3-V2D-CPB	70.6	45.3	5656
1.5-V1D-CPB	75.3	65.1	6020
2.1-V1-OSB	74.6	69.8	3298
2.3-V2D-OSB	76.0	70.4	4851

## Data Availability

The data presented in this study are available on request from the corresponding author.
